# Implementation of Integrated Care of Patients with Non-Cancer Chronic Pain in Chile: Advances and Challenges

**DOI:** 10.5334/ijic.8970

**Published:** 2025-04-28

**Authors:** Paula Zamorano, Teresita Varela, Isidora Salvatierra, Leonardo Silva, Víctor Lucero, Denisse Figueroa, Sheila Salazar, Pia Casas, Nidia Castro, Pablo Montecinos, Francisco Salinas

**Affiliations:** 1 Innovación ANCORA UC, Facultad de Medicina, Pontificia Universidad Católica de Chile, Santiago, Chile; 2 Escuela de Ciencias de la Salud, Pontificia Universidad Católica de Chile, Santiago, Chile; 3 Departamento de Medicina Familiar, Facultad de Medicina, Pontificia Universidad Católica de Chile. Santiago, Chile; 4 Centro de Salud Familiar La Florida, Corporación Municipal de La Florida, Chile; 5 Centro de Salud Familiar Maffioletti, Corporación Municipal de La Florida, Chile; 6 Centro de Salud Familiar Segismundo Iturra, Dirección de Salud de San Felipe, Chile; 7 Corporación Municipal Gabriel González Videla de La Serena, Chile

**Keywords:** noncancer chronic pain, patient-centered care, primary care, dolor crónico no oncológico, atención centrada en el paciente, atención primaria

## Abstract

The impact of chronic non-cancer pain on individuals is complex, multidimensional, and with healthcare needs that challenge fragmented healthcare systems. There have been significant advances in generating scientific evidence, ministerial guidelines, and even laws in Chile. However, there is still a considerable gap that remains in the implementation in clinical practice. Despite this, clinical teams have implemented three pilot programs in different countries. This perspective discusses these pilots, their implementation process, acquired knowledge, and the challenge of advancing toward scaling up.

## Abstract (es)

El impacto del dolor crónico no oncológico en las personas es complejo y multidimensional cuyas necesidades de atención clínica desafían a los sistemas de salud fragmentados. En Chile ha habido avances significativos en la generación de evidencia científica, lineamientos ministeriales e incluso leyes. Sin embargo, todavía existe un vacío considerable en su implementación en la práctica clínica nacional. Por ello, desde los equipos clínicos han sido pioneros implementado tres pilotos en diferentes regiones. Esta perspectiva analiza estos pilotos, su proceso de implementación, los conocimientos adquiridos y el desafío de avanzar hacia su escalamiento.

## Introduction

Chronic non-cancer pain (CNCP) has become a global public health issue [1] due to the magnitude of the problem and its consequences for individuals, families, society, and the healthcare system. The impact on individuals is multidimensional, affecting them physically (functionality), emotionally, within their family, socially, and in their work life, among other areas. In Chile, it is one of the most common reasons for clinical consultations, with a prevalence ranging from 27% to 34%, being higher among those with lower socioeconomic status and older age [2,3]. The cost represents 0.32% of the GDP and 5% of healthcare costs [4]. Additionally, the healthcare system’s response is fragmented and insufficient, relying mainly on medical care and pharmacological treatment, which is not enough to address the multidimensional impact of this health problem.

In Chile, in 2013, cost [5], prevalence [6] and impact of resource consumption and labor productivity of the CNCP [7] have been evaluated, and a public policy proposal for managing chronic musculoskeletal pain was developed in 2018. In 2020, the Ministry of Health initiated the implementation of a Person-Centered Comprehensive Care Strategy (ECICEP) [8] and issued technical guidelines for the management of chronic non-cancer pain in individuals aged 15 and older in primary healthcare (PHC) in 2021 [9]. Subsequently, in 2023, Law N° 21.531 on Fibromyalgia and other chronic non-cancer pain was enacted. However, applying these clinical practices has been slow and limited, relying on local initiatives that have disposed of their resources to meet this need.

This article addresses three pilot programs in different regions of the country that exemplify a “bottom-up” [10] implementation and are currently serving as learning references for the Ministry of Health.

## Approach to People with chronic non-cancer Pain in clinical practice

The first pilot started in 2019 when the Pontificia Universidad Catolica of Chile (PUC) and the Municipal Corporation of La Florida started a partnership to implement a pilot intervention in two primary care centers (La Florida and Maffioletti) in collaboration with a high-complexity hospital (Hospital La Florida) in the Metropolitan Region. The studied individuals aged 25 to 60 with fibromyalgia, painful shoulder syndrome, low back pain, osteoarthritis, and rheumatoid arthritis. The aim is to improve quality of life through strategies that strengthen continuity of care, diversify services, and enhance interdisciplinary work. The clinical intervention includes a comprehensive evaluation within ECICEP, physiotherapy in pain neuroscience, pain psychology, and complementary pharmacology [11]. In addition to direct clinical activity, periodic clinical consultations were performed with clinical teams and the physiatry team at La Florida Hospital, postgraduate courses offered by PUC, technical reinforcement workshops, clinical modeling, and academic support for health professionals. Practical guides have been developed for group and individual clinical activities in physiotherapy, psychology, and nutrition. The pilot proposes an evaluation based on the impact on health quality of life, health services utilization, drug consumption, and productivity losses.

The advances to date have provided operational learnings, articulation of internal flows, and performance, and revealed cultural change management challenges. As of May 2024, nearly 600 patients have accessed the services. Upon the end of the pilot, if quality of life improves, then the municipality will continue with the clinical intervention as part of their standard care.

The second pilot began in 2022 at the Segismundo Iturra Family primary care center in the San Felipe municipality, located in the northern part of the country. The exposed population was adults with widespread or localized chronic pain, including severe knee and/or hip osteoarthritis without surgical indication, chronic low back pain, and fibromyalgia. Patients wishing to access the intervention must have active support networks, show interest, and have time availability. The objective is to provide access and opportunity for comprehensive, multimodal, person-centered treatment. Approximately 200 people have received the intervention, of which 90% are women and 10% are men. The clinical intervention includes pre-admission interviews conducted by psychologists and speech therapists, medical interviews where a comprehensive consensus-based plan is developed, and access to pharmacological treatment and rehabilitation workshops with an occupational therapist and a physiotherapist. Clinical emphasis is on exercises, relaxation techniques, routine structuring, and self-care. They can also access the *SER* Human Development and Wellness Center for integrative physiotherapy, complementary therapies, and health wellness practices. The implementation process has involved reorganizing resources and redesigning healthcare processes to enable the transition from prescriptive, disease-centered care to a collaborative, person-centered, and self-management-focused approach.

The progress to date has allowed the consolidation of the intervention in this health care center, coordinated with the *SER* Center and secondary-level care (San Camilo Hospital). Integrating interdisciplinary services such as mental health, nutrition, nursing, and social support positions this pilot as a model for humanization and holistic care. The pilot evaluation is proposed in terms of adherence and pain perception. In contrast to the first pilot, San Felipe has decided that this intervention will have continuity as part of their health services for adult patients.

The third pilot began in 2023 in the La Serena municipality, located in the Coquimbo Region (IV Region), at six primary health centers. Their studied population is adults diagnosed with chronic non-cancer pain who have been referred to community rehabilitation. This intervention aims to address pain management through physiotherapy, multidisciplinary education, and self-management tools based on group therapy to promote social and community integration. Two hundred users have participated, with 95% women and 5% men. Regarding medical diagnoses, 83% have been diagnosed with fibromyalgia, 10% with rheumatoid arthritis, and 7% with other chronic pain conditions. The clinical intervention is based on a transdisciplinary approach to pain, involving various healthcare professionals, such as pharmacists, psychologists, and nutritionists. Group educational and therapeutic exercise sessions are offered, including physiotherapy, complementary therapies, education by social workers, tai chi classes, hydrotherapy, and craft workshops. The implementation process has enabled this pilot to be linked with existing preventive national strategies such as the “Elige Vivir Sano” Program and the “MAS adultos mayores autovalentes” Program.

Progress to date has shown that pain perception and functional limitations have been reduced, emotional pain management has improved, and peer connections have been established. The evaluation will focus on the reduction of waiting times, optimization of health resources, and promotion of self-management through the formation of community groups. As the second pilot, this intervention will continue as part of the standard care once the local context adjustments are completed.

## Conclusion and recommendations

The progress of these three pilots in addressing adults with CNCP in the public health system in Chile demonstrates advances such as, first, that implementation in a real-world context is feasible, where the humanization of healthcare through interdisciplinary/transdisciplinary person-centered interventions is essential. Second, their implementation depends on the ability to reorganize existing resources and incorporate services that respond to the impact of CNCP, creating variability across regions that also reflect the diversity of their populations. Third, the magnitude of patients’ needs is directly perceived by healthcare teams and drives complex changes in the system, making it essential to include change management. Fourth, bottom-up implementation has been possible with the motivation of the clinical teams and the ministerial technical orientation, which shows that these three experiences have similar intervention strategies. Their learning has been shared within healthcare teams, governance, and academics in publications, national seminars, and international congresses to advocate for those who need it. Still, the scientific evidence on the impact on health system performance and patient outcomes, minimum conditions, and resources needed should be the product of these pilots to facilitate the national scaling-up from the Ministry of Health.
